# Re-Annotator: Annotation Pipeline for Microarray Probe Sequences

**DOI:** 10.1371/journal.pone.0139516

**Published:** 2015-10-01

**Authors:** Janine Arloth, Daniel M. Bader, Simone Röh, Andre Altmann

**Affiliations:** 1 Translational Research Department, Max Planck Institute of Psychiatry, Kraepelinstrasse 2–10, 80804, Munich, Germany; 2 Gene Center Munich, Ludwig-Maximillians-Universität München, Feodor-Lynen Strasse 25, 81377, Munich, Germany; 3 Department of Neurology and Neurological Sciences, Stanford University, School of Medicine, 780 Welch Road, CJ350 C38, CA-94304 Palo Alto, California, United States of America; Huazhong University of Science and Technology, CHINA

## Abstract

Microarray technologies are established approaches for high throughput gene expression, methylation and genotyping analysis. An accurate mapping of the array probes is essential to generate reliable biological findings. However, manufacturers of the microarray platforms typically provide incomplete and outdated annotation tables, which often rely on older genome and transcriptome versions that differ substantially from up-to-date sequence databases. Here, we present the *Re-Annotator*, a re-annotation pipeline for microarray probe sequences. It is primarily designed for gene expression microarrays but can also be adapted to other types of microarrays. The Re-Annotator uses a custom-built mRNA reference database to identify the positions of gene expression array probe sequences. We applied Re-Annotator to the Illumina Human-HT12 v4 microarray platform and found that about one quarter (25%) of the probes differed from the manufacturer’s annotation. In further computational experiments on experimental gene expression data, we compared Re-Annotator to another probe re-annotation tool, ReMOAT, and found that Re-Annotator provided an improved re-annotation of microarray probes. A thorough re-annotation of probe information is crucial to any microarray analysis. The Re-Annotator pipeline is freely available at http://sourceforge.net/projects/reannotator along with re-annotated files for Illumina microarrays HumanHT-12 v3/v4 and MouseRef-8 v2.

## Introduction

Analysis of gene expression profiles under various conditions is one of the corner stones in modern molecular biology research. One major challenge in working with gene expression microarrays is the quality of the annotation of the array probes used by the platform. Differences in probe annotations complicate the replication of studies as well as meta-analyses across platforms. Moreover, the annotations provided by the manufacturers quickly become outdated with every update of the genome assemblies as well as the accompanying annotation tables. For example, the number of annotated transcripts in the RefSeq Gene database (RefSeq release 59 [1] differs from hg18 (NCBI build 36.1) to hg19 (GRCh37 build 37) assembly by more than 1,200 transcripts (43,236 to 44,596).

Furthermore, the initial mapping provided by the manufacturers contains several severe problems–some probes map to non-transcribed genomic regions, bind secondary targets or have other properties that may confound a proper analysis, such as common SNPs in the probe sequence. Clearly, these probes should be removed from the analysis as an accurate probe annotation is fundamental for all downstream analyses and ensures accurate biological interpretation of the results. Outdated annotation of probes becomes an increasing problem in publicly available gene expression catalogues such as the ALLEN brain atlas [2] as researchers tend to use the provided expression data as is, that is, without further validity checks and quality control.

In order to identify probes with potential annotation problems, a sound re-annotation of all probes is required. Recently, approaches were developed that allow the re-annotation of gene expression microarray data by re-aligning the probe sequences to the entire human genome [3,4]. However, when using the whole genome as the mapping reference, there is an increased likelihood of short reads being mapped to multiple locations and intergenic region and thereby decreasing the number of uniquely mappable probes [5]. Still, 24% of the human genome cannot be uniquely mapped using 50 bp long sequences with two mismatches [6,7], which corresponds to the sequence length of Illumina array probes. Therefore, including untranscribed regions, which theoretically should not even be part of the cDNA library of interest, reduces the mappability and introduces an additional source of unnecessary errors.

Our approach, Re-Annotator, considers these mappability issues: in a first round the pipeline maps microarray probe sequences directly to a custom-built mRNA reference instead of the entire human genome sequence. Thus, our pipeline enables us to correctly annotate various formerly non-mappable probes [5].

In the following we describe the Re-Annotator pipeline and use it to re-annotate the probes sequences of two Illumina gene expression microarrays: one for human (HumanHT-12 v4) and one for mice (MouseRef8 v2). In further computational experiments we compare the re-annotation by Re-Annotator and a genome only based re-annotation by ReMOAT [3] for experimental gene expression data.

## Materials and Methods

### The Re-Annotator Pipeline

The pipeline comprises two essential parts: (1) the creation of a customized reference sequence database and (2) the step-wise re-annotation of the probe sequence.

#### *In Silico* mRNA reference database

**Fig 1:**
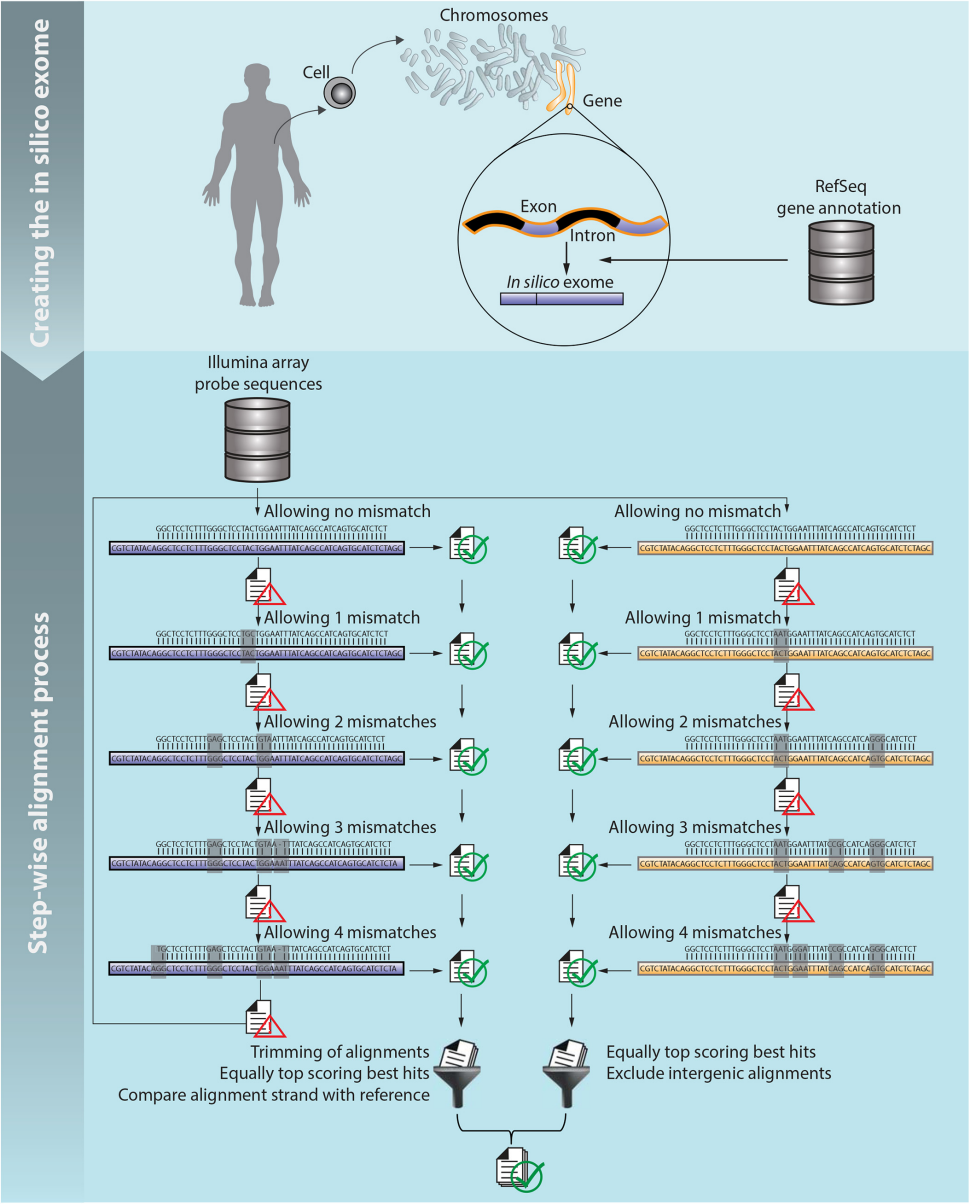
Annotation pipeline. Schematic overview of the computational pipeline flow: (top) creating the *in silico* mRNA reference database and (bottom) step-wise alignment process. Purple sequences in the left column correspond to alignments to the *in silico* reference and brown sequences in the right column correspond to alignments to the genome.

First, for each transcript in the RefSeq database the genomic location of the exons are extracted. Next, the genomic sequence of the exons of each isoform are concatenated to form one *in silico* mRNA. These sequences (one per listed isoform in the database) form the reference database for the mapping step (Fig 1).

#### Step-wise alignment process

The annotation process starts with the alignment of the probe sequences provided by the manufacturer to the mRNA reference database using the BWA [8]. The quality of the probe sequence does not decrease by position (as it would with real sequencing data), thus the aligner is executed without seeding (seed length > length of probe sequence). A step-wise alignment process is performed allowing at first no mismatches between probe sequence and reference sequence. Next, all unaligned probe sequences are resubmitted for alignment with the number of allowed mismatches increased by one. Array probe sequences that do not align to the mRNA reference database with a maximum of four mismatches are mapped to the whole genome using the same step-wise approach. Alignments are stored in SAM format [9] where all alternative best hits are included. Successful alignments to the mRNA reference database are processed according to the following steps:

1. Alignments starting or ending with a mismatch, insertion or deletion are trimmed
2. Alignment positions in the *in silico* mRNA are mapped to the genome position
3. Alignments are discarded if the alignment strand does not match the original coding strand information of the RefSeq database

Likewise, successful alignments to the genome sequence are processed according the following steps:

1. Steps (1) and (3) of mRNA reference alignments procedure above
2. Gene annotation is added using the genome annotation tool ANNOVAR [10]
3. Alignments that are annotated to be in intergenic or up- and downstream regions (defined as 1 kb distance to the transcription start or end site) are excluded

After these steps for each mapped probe we know its genomic positions and which genes in the RefSeq database are located at the probe’s genomic location. Optionally, the location of SNPs in mapped probe sequence can be added to the annotation.

#### Annotation post-processing

In order to ensure that a probe is specific for one genomic region, we eliminate probes with multiple hits that cannot be assigned to the same gene, i.e., we recommend that coordinates of different hits should be not more than 25 bp apart (default for Illumina arrays) from each other. This threshold is largely arbitrary (half of the probe length of Illumina probes) and it may remove a few relevant probes, but it will guarantee that the remaining probes are aligned uniquely to a distinct region. Further, we require that probes with multiple annotations should be aligned in the same direction (alternate haplotype regions of the original assembly were ignored). The final re-annotation is provided with additional information on the probe gene symbol and updated position (also for splice-annotations).

#### System requirements

Re-Annotator comprises shell and Perl scripts. In order to successfully execute the Re-Annotator pipeline following software should be pre-installed on the system: the Burrows-Wheeler Aligner (BWA) [8], SAMtools [9], ANNOVAR [10] and Perl. In addition, following databases should be available: the genome assembly of interest (e.g., hg19), a corresponding gene annotation table (e.g., RefSeq), the microarray probe sequences to be annotated and optionally a SNP database (e.g., dbSNP or 1000 genomes).

### Re-Annotation of Illumina Gene Expression Microarrays

We applied the Re-Annotator pipeline to the probe sequences of Illumina HumanHT-12 v4 (http://support.illumina.com/array/array_kits/humanht-12_v4_expression_beadchip_kit/downloads.html) and Human MouseRef-8 v2 (http://support.illumina.com/array/array_kits/mouseref-8_v2_expression_beadchip_kit/downloads.html). As a reference genome we used hg19 and mm9, respectively, obtained from the UCSC Genome Browser [11]. The mRNA reference databases were built on the RefSeq database versions provided by the UCSC Genome Browser (downloaded January 2012). Furthermore, we used the SNPs for the central European population (CEU) from the 1000 genomes database [12] for annotating SNPs in mapped probe sequences.

### Competitive Comparison of Re-Annotator on Experimental Gene Expression Data

We re-annotated the probe sequences of the HumanHT-12 v4 chip with ReMOAT [3] another re-annotation software in order to compare Re-Annotator to a genome only re-annotation approach. ReMOAT is based on a BLAST search against the corresponding genome and all transcripts defined in the UCSC annotation tables. For this head-to-head comparison we consider “good” probes to be rated as “good” or “excellent” by ReMOAT and probes with a uniquely identified gene by Re-Annotator; “bad” probes are the remaining probes. Further, we used experimental data to quantify the differences between different annotations and to highlight the effects of different annotations on experimental results.

#### Experimental data and pre-processing

We reanalyzed gene expression profiles in whole blood cells from 36 individuals at baseline (i.e., without any stimulation) hybridized to the Illumina HumanHT-12 v4 microarray chips (Gene Expression Omnibus Accession: GSE64930). First, each probe was independently filtered using a detection *p*-value of 0.01 in at least 18 subjects (50%), leaving 13,610 expressed probes for further analysis. Secondly, intensity values were transformed and normalized through variance stabilization and normalization (VSN) [13].

#### Comparison of Re-Annotator and ReMOAT

We compared Re-Annotator and ReMOAT using two different measures. The first measure examines the variance of probes in different groups and the second measure is concerned with whether probes passed the detection threshold or not.

Using the real-world dataset we grouped the probes in five categories. Categories 1 and 2 served as reference and comprise probes that were annotated as “good” and as “bad” by both tools, respectively. We refer to category 1 and 2 as “Both” and “None”, respectively. Category 3 comprised probes that were annotated as “good” by Re-Annotator and as “bad” by ReMOAT, while category 4 comprised probes that were annotated as “bad” by Re-Annotator and as “good” by ReMOAT. Categories 3 and 4 are referred to as “InRA” and “InRM”, respectively. Category 5 comprised probes that are marked as repeats by ReMOAT using RepeatMasker (www.repeatmasker.org) (“InRepeat”). We compared the mean relative variability (coefficient of variation = CV) of probes in categories 3–5 to the mean CV in both reference categories 1 and 2 using a two-sided Wilcoxon test.

For the second measure we hypothesized that probes annotated as “bad” are less likely to pass the detection criteria, i.e., they are more likely considered not expressed in the experiment. For each annotation tool we computed the 2x2 contingency table between annotation (“good” and “bad”) and expression status (“expressed” and “not expressed”) and derived the odds ratio (OR) along with the 95% confidence interval (CI). Next, we compared the ORs for Re-Annotator and ReMOAT.

## Results

### Re-Annotation of Illumina Gene Expression Microarrays

#### Re-Annotator improves annotation of human probes compared to manufacturer

**Fig 2:**
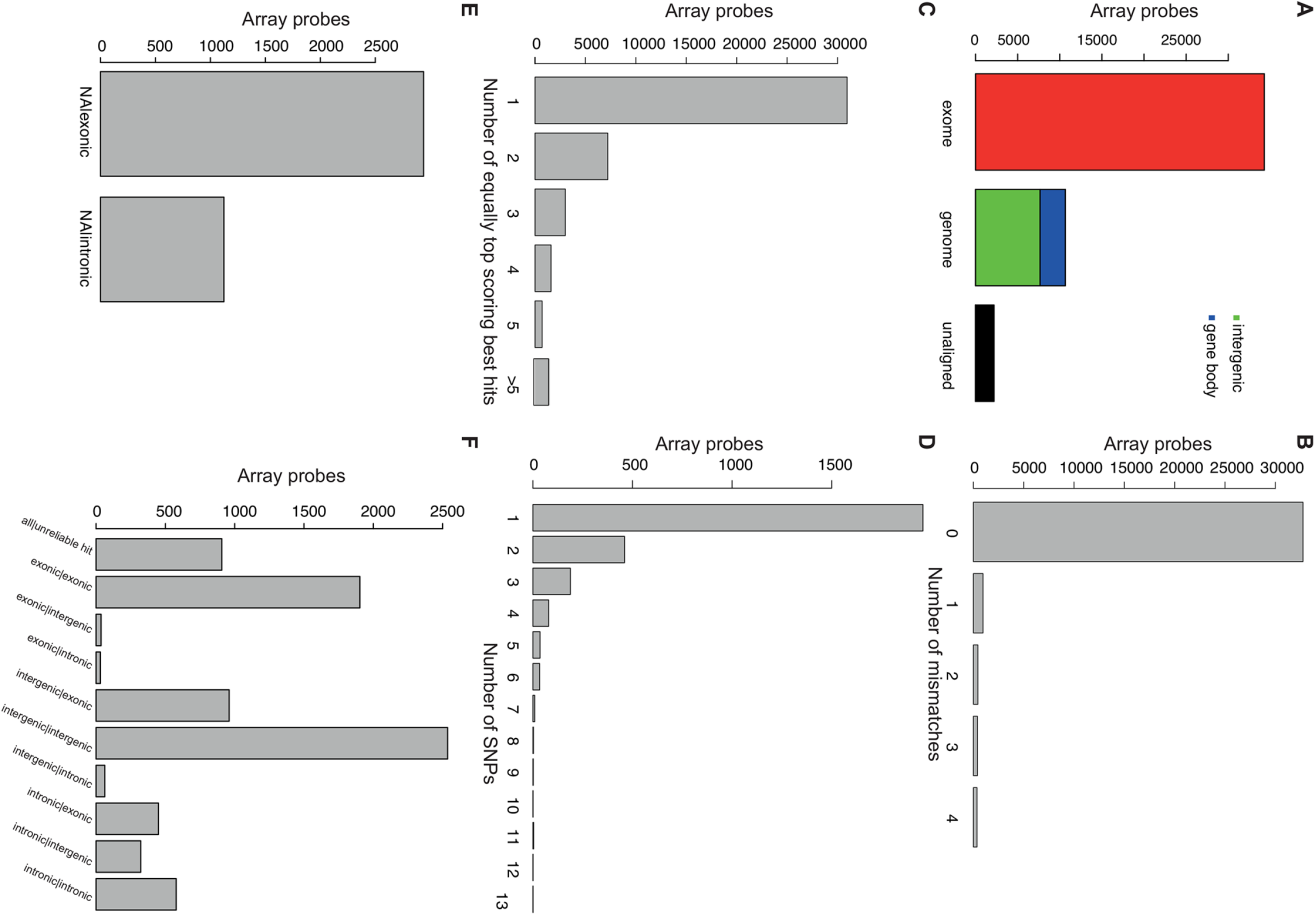
Results of the re-annotation of Illumina HumanHT-12 v4 probe sequences. (A) Barplot of the alignment basis. The left bar represents the array probe sequences that could be aligned to the *in silico* mRNA reference database. The middle bar represents sequences that were aligned to the whole genome reference subdivided into genic and intergenic alignments. The right bar represents unaligned sequences. Histograms showing (B) the number of mismatches between probe sequence and reference, (C) the number of equally top scoring best hits per probe sequence and (D) the number of SNPs (in the 1000 genomes data) within an aligned probe sequence. (E) Histogram of the annotation of probes, which have no annotation according to the manufacture and now have been rescued and reliably annotated. (F) Histogram showing the changes in annotation from the manufacture to our re-annotation (Manufacture | Re-Annotator).

We analyzed the Illumina HumanHT-12 v4 probe sequences using the Re-Annotator Pipeline. Of all 47,230 probe sequences, 95% (Fig 2A; Table 1) were aligned to either our custom-built mRNA sequence database (*n* = 34,277; in the first alignment step) or if no hit was found to the reference genome (*n* = 10,661; in the second alignment step). A large fraction of the latter probe sequences were aligned to genomic locations without any known transcribed gene (*n* = 7,493). After the post-processing filter, 77.7% of all aligned probe sequences (see Table 1) mapped to a distinct region (defined as a maximum of 25 bp distance between multiple hits for the same sequence) in the genome and were included in the final annotation file for the HumanHT-12 v4 BeadChip array. This set of 34,936 probes is referred to as “*reliable*” array probes in the following. The majority (93.8%) of those reliable probes (Fig 2B; Table 1) were aligned without mismatches. The number of hits per probe to a region ranged from 1 to 32, where 67.7% had only one unique hit and 96% (*n* = 33,539) had less than five hits (Fig 2C). The vast majority of reliable probes (92.1%; Fig 2D; Table 1) resided in regions without known SNPs in the Caucasian population (based on the 1,000 Genomes Project). It is conceivable that SNPs within the probe sequence may be the source of “differential” expression via altered hybridization efficiency. However, Schurmann et al. [4] reported no consistent effects of SNPs located in array probe sequences on hybridization efficiency. Thus, one has to test individually whether these SNPs are associated with alternate expression signals intensity.

**Table 1:** Number of probe sequences at different steps of the re-annotation for two Illumina chips. Detailed numbers for probes tat could be aligned (Aligned), that were considered “good” probes after the post-processing, and were aligned without mismatches to the reference genome, or were unique alignments.

	HumanHT-12 v4	MouseRef-8 v2
**Total**	47,132	25,697
**Aligned**	44,938	25,542
**Post-processing (“good”)**	34,936	24,799
**No mismatch**	32,754	NA
**Single alignment hit**	23,661	23,187

Roughly 23.5% (n = 11,086) of all Illumina probes were not annotated with probe coordinates and gene symbols by the manufacturer. Re-Annotator rescued and reliably annotated about 36,6% (*n* = 4,062) of these previously un-annotated probes. Seventy-two percent (n = 2,939) of these probes were annotated to exonic regions and the rest to intronic regions (see Fig 2E). A total of 36,144 probes had a complete Illumina annotation. Re-Annotator provided an annotation differing from the manufacturer’s for 21.5% (n = 7,789) of these probes (see Fig 2F). Thus in summary Re-Annotator provided an updated annotation for one quarter (25%) of all probes on the Illumina HumanHT-12 v4 chip.

#### Re-Annotator refines annotation of mouse probes compared to manufacturer

**Fig 3:**
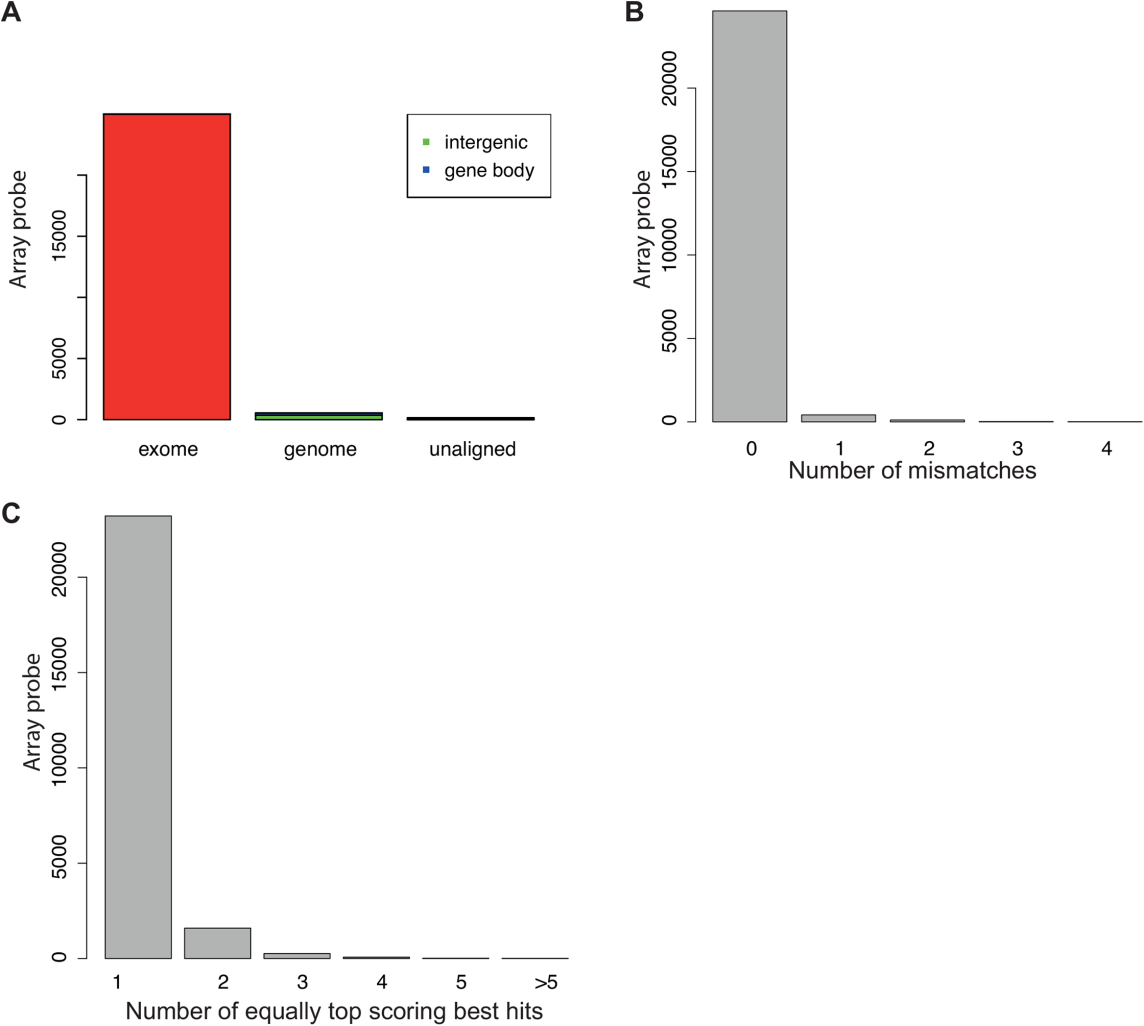
Results of the re-annotation of Illumina MouseRef-8 v2 probe sequences. (A) Barplot of the alignment basis. The left bar represents the array probe sequences that could be aligned to the *in silico* mRNA reference database. The middle bar represents sequences that were aligned to the whole genome reference subdivided into genic and intergenic alignments. The right bar represents unaligned sequences. Histograms showing (B) the number of mismatches between probe sequence and reference and (C) the number of equally top scoring best hits per probe sequence.

Additionally, we analyzed the Illumina MouseRef-8 v2 probe sequences (*n* = 25,697) using our Re-Annotator pipeline. Almost all probes were aligned (99.4%; Fig 3A; Table 1) to either the mRNA reference database (*n* = 24,994) or the genome sequence (*n* = 548) and passed the post-processing filter (Table 1). 97.9% of all post-processed array probe sequences were aligned without mismatches (Fig 3B; Table 1) and 93.5% mapped to a single region with only 14 probes having five or more hits per probe (Fig 3C; Table 1). The successful re-annotation of the mouse microarray can be explained by the reduced array content to only RefSeq genes, providing good transcriptomic annotation quality.

### Competitive Comparison of Re-Annotator on Experimental Gene Expression Data

**Fig 4:**
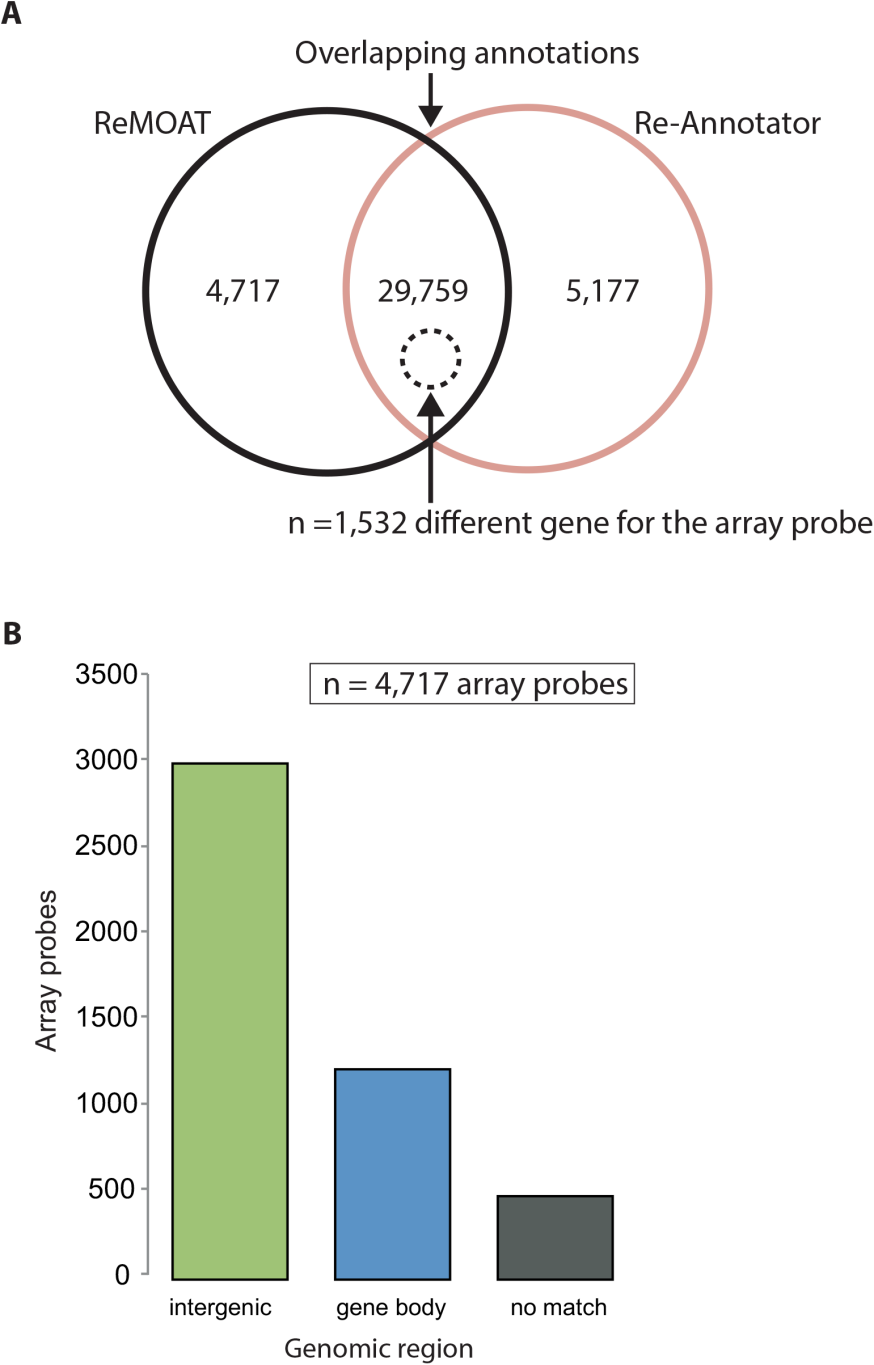
Comparison between Re-Annotator and ReMOAT on HumanHT-12 v4 probe sequences. (A) Venn diagram representing the overlap of transcripts annotated with ReMOAT and Re-Annotator. A total of 5% of the probe sequences were annotated with different genes by Re-Annotator and ReMOAT. (B) Bar graph detailing the exclusion reason for probes included by ReMOAT but excluded by Re-Annotator.

Comparing Re-Annotator to ReMOAT, another re-annotation tool, we found that 86.3% (*n* = 29,759) of probe sequences annotated as "good" by ReMOAT (quality equal to”Perfect” and “Good”; *n* = 34,476 sequences) were also classified as “good” probes by Re-Annotator (Fig 4A). However, 5% (*n* = 1,532) of these 29,795 array probes received different annotations by the two tools. A total of 4,717 probes, which were annotated as “good” by ReMOAT, were excluded by Re-Annotator due to alignments in intergenic regions (63.7%), multiple different hits in the mRNA reference (26%) and no alignment (10.3%) (Fig 4B).

#### Re-Annotator provides probes with less variable intensity then ReMOAT

We computed the coefficient of variance (CV) for each probe across all 36 subjects and compared the mean CV of the probes in the categories InRA, InRM and InRepeat to categories Both and None. Table 2 lists the group sizes (number of probes within a group) of the five categories for all expressed probes along with the estimated CV (mean and standard deviation). InRA has a significantly reduced CV compared to “None” (P = 0.002, two-sided Wilcoxon test) but not to “Both” (P = 0.69, two-sided Wilcoxon test). The reverse holds true for InRM (P = 9.58x10^-6^ for “Both”; P = 0.85 for “None”, two-sided Wilcoxon test). Suggesting that probes retained only by Re-Annotator behaved more like probes retained by both tools than probes retained only by ReMOAT. Further, our experiment showed that probes in repeat regions displayed an unchanged CV compared to “Both” (P = 0.17 two-sided Wilcoxon test) but a significantly reduced CV compared to “None” (P = 9.82x10^-5^, two-sided Wilcoxon test).

**Table 2:** Group size and relative variability for the five probe categories. Rows three and four compare the variance for probes in categories InRA, InRM and InRepeat to the two reference categories Both and None, respectively, using a two-sided Wilcoxon test.

	Both	None	InRA	InRM	InRepeat
**Number of probes**	9,236	377	1,208	433	1,176
**Mean CV (SD)**	0.1709 (0.002)	0.1714 (0.003)	0.1711 (0.002)	0.1716 (0.005)	0.1709 (0.002)
**P-value (Wilcoxon test statistic) wrt. None**	NA	NA	0.0026 (W = 204,340)	0.85 (W = 82,264)	9.82 x10^-5^ (W = 192,160)
**P-value (Wilcoxon test statistic) wrt. Both** ^a^	NA	NA	0.69 (W = 736,370)	9.58x10^-6^ (W = 110,040)	0.17 (W = 668,970)

^a^For the test we drew a subsample of the “Both” group to match the sample size of the corresponding comparison group.

#### Re-Annotator provides probes that are more likely to be expressed

Table 3 lists the two 2x2 contingency tables for expression status and annotation by the two tools. Based on these values, probes annotated as “bad” were less likely to be expressed. However, the effect was significantly stronger for Re-Annotator (OR: 3.9; 95% CI: 3.65–4.16) than for ReMOAT (OR: 2.11; 95% CI: 2.01–2.23) (P<0.05; due to lack of overlap in the CIs).

**Table 3:** Contingency tables between expression status and annotation for Re-Annotator and ReMOAT.

	Re-Annotator		ReMOAT	
	Good	Bad	Good	Bad
**Expressed**	12,321	1,227	11,431	2,117
**Not Expressed**	22,615	8,775	22,559	8,831

## Discussion

**Fig 5:**
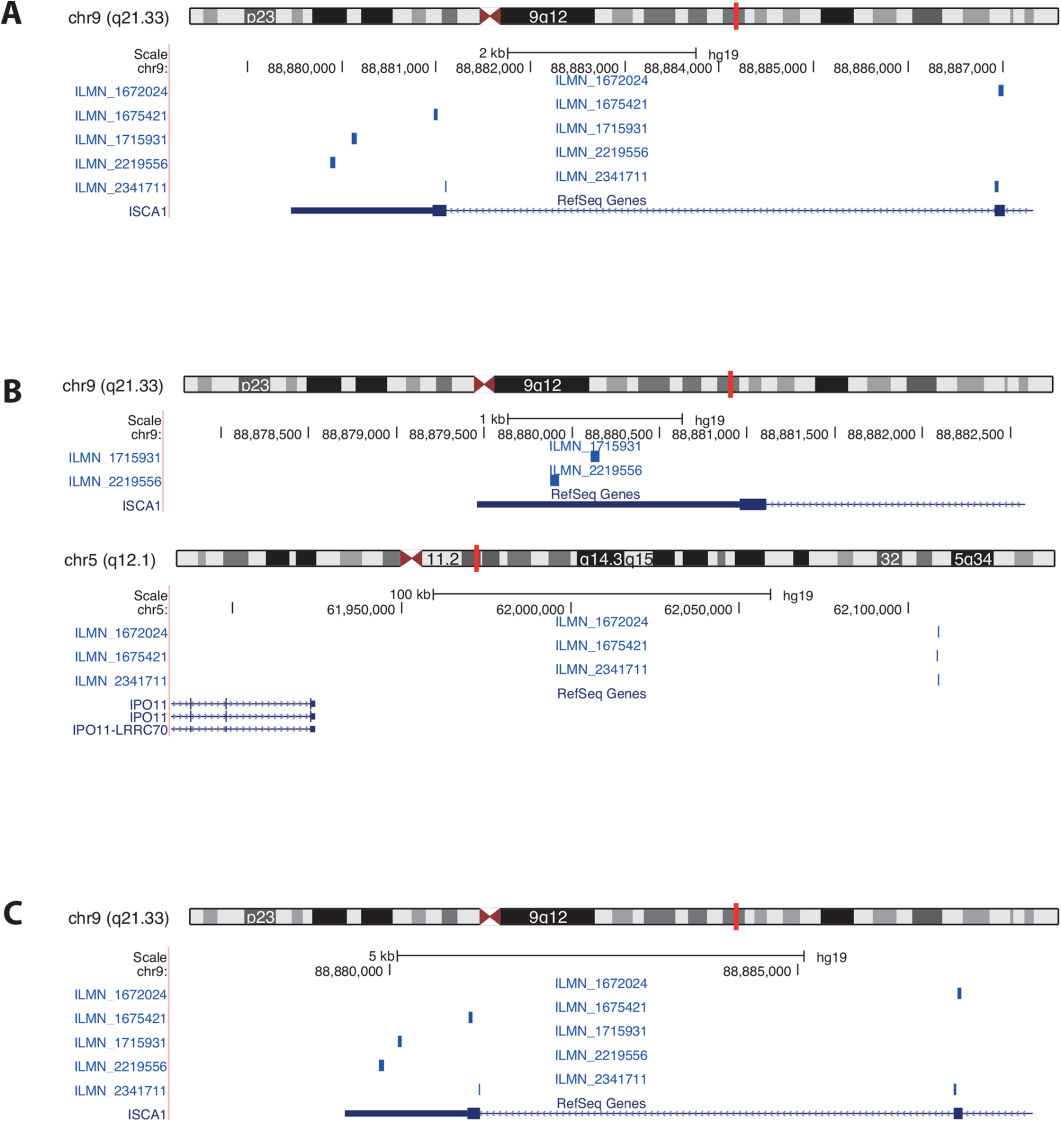
USCS genome browser graphic for the human ISCA1 gene. The gene is located on chromosome 9; the targeting Illumina probes are ILMN_1715931, ILMN_1672024, ILMN_2219556, ILMN_1675421 and ILMN_2341711. Custom tracks represent the probe sequences annotated by (A) the Re-Annotator, (B) manufacturer and (C) ReMOAT.

**Fig 6:**
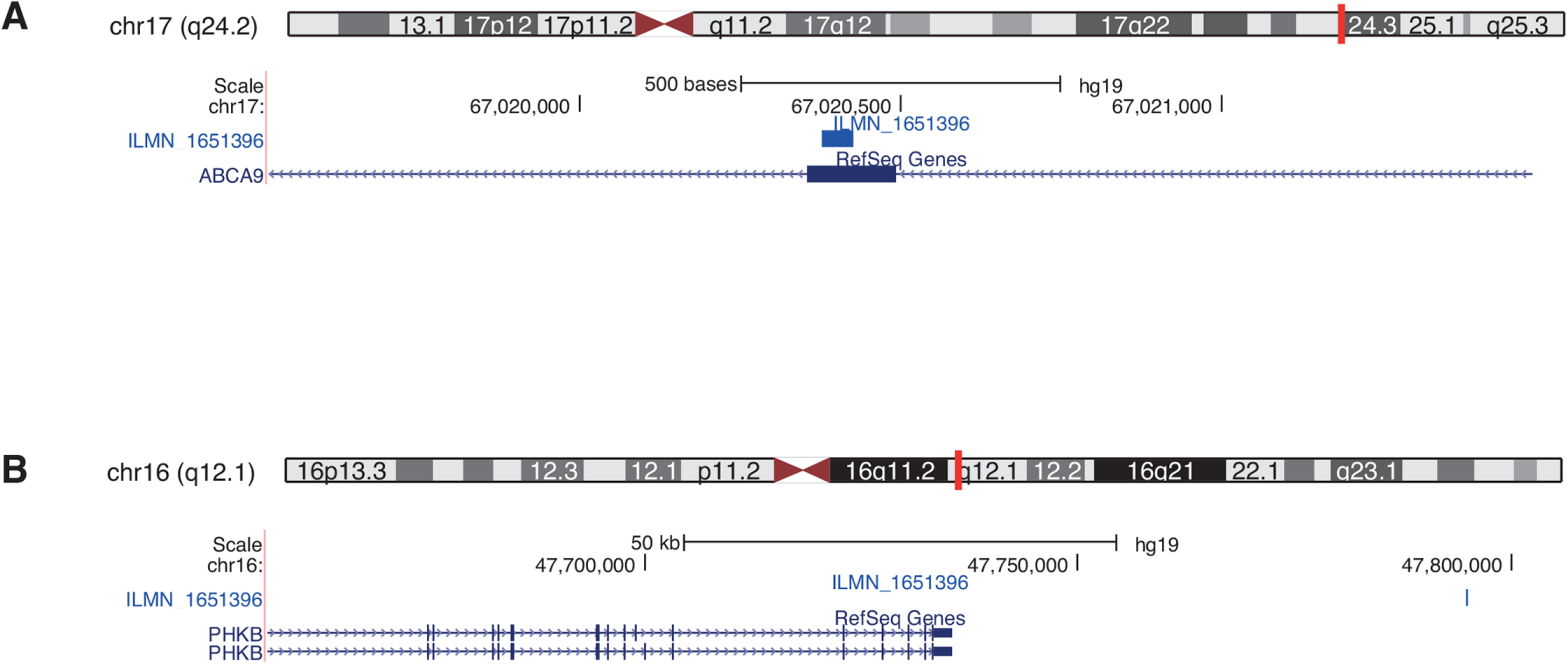
USCS genome browser graphic for the human ABCA9 gene. The gene is located on chromosome 9; the targeting Illumina probe is ILMN_1651396. Custom tracks represent the probe sequences annotated by (A) the Re-Annotator and (B) manufacturer and ReMOAT.

A precise annotation of microarray probe sequences is essential for accurate biological findings and replicability. In this work, we present a pipeline to re-annotate probe sequences of gene expression microarrays using a custom-built mRNA reference and applied it to three Illumina BeadChip arrays (Human HT-12 v3, v4 and MouseRef-8 v2). The re-annotation revealed that indeed one quarter of the array probes were incompletely or incorrectly annotated by the manufacturer. A source of such mis-annotation may be due to changes in genome assembly or changes in exon/intron boundaries since the original design of the chip. Over 21% of re-annotated probes were assigned to different genes as given by the manufacturer. For example, three of the five Illumina HumanHT-12 v4 array probe sequences illustrated in Fig 5 all perfectly re-annotated within the first or second exon of the human gene ISCA1 on chromosome 9 using the Re-Annotator. Originally these probes were annotated on chromosome 5 within an intergenic region (Fig 5). A reason for this discrepancy was that the probe sequences were designed using an older assembly version (hg18). In this release, the region on chromosome 5 was annotated with the gene ISCA1L. The three probes, however, also have a perfect match on chromosome 9 in the ISCA1 gene. In the new release (hg19), the ISCA1L gene was removed, i.e., the region on chromosome 5 is without annotation, and therefore Re-Annotator selected the region on chromosome 9 in the ISCA1 gene. Hence, it is important to keep the annotation tables of the probes up-to-date. ReMOAT, based on a genomic alignment, placed these probes in accordance with the Re-Annotator annotation (Fig 5). We recommend checking all given probe sequence annotations (second matches as well as other given genomic matches) also when using the ReMOAT annotation, as the given genomic location might be incorrect. Such an example is illustrated in Fig 6; the probe sequence was allocated to an intergenic region. We annotated this probe sequence to be on chromosome 17 within an exon of ABCA9, which was in accordance with the second match of the ReMOAT annotation.

Furthermore, the Re-Annotator conducts no filtering based on the RepeatMasker as recommended by Barbosa-Morais et al. [3]. However, we found many regions marked by this algorithm to be perfectly mappable, and filtering may eliminate data on important genes. An example is a probe located within the FKBP5 gene (ILNM_1778444). When applying repeat masking, this probe is marked as unreliable since it is located within a short interspersed nuclear element (SINE). Still, there are no issues of mappability or uniqueness; thus, the probe should not be excluded from further analysis.

Approximately 74% of all human probes present on the latest Illumina gene expression array (HumanHT-12 v4) were uniquely allocated to one gene locus. Such a re-annotation is important for removing uninformative probes, such as probes that cannot be placed into a distinct region, before starting differential gene expression analysis. This increases specificity of an analysis and will decrease the false discovery rate. With our pipeline we closed these gaps and compensated for wrong annotations.

A thorough re-annotation of probe sequences is not a standard part of gene expression microarray analysis. To highlight its profound effect, we applied our pipeline to Illumina BeadChip Human HT-12 v4 and compared it to the Illumina annotation as well as to the ReMOAT annotation. We discovered that the Human HT-12 v4 re-annotation differs substantially from the annotations provided by Illumina and ReMoat (by 25% and 16%, respectively). Our pipeline improves the probe annotation and proves to be an essential step in producing high quality microarray results.

The Re-Annotator pipeline is freely available at http://sourceforge.net/projects/reannotator along with re-annotated files for Illumina microarrays HumanHT-12 v3/v4 and MouseRef-8 v2.
